# Patients’ and Parents’ Perceptions of Appearance in Scoliosis Treated with a Brace: A Cross-Sectional Analysis

**DOI:** 10.1007/s10826-013-9776-4

**Published:** 2013-07-23

**Authors:** Ewa Misterska, Maciej Glowacki, Katarzyna Adamczyk, Roman Jankowski

**Affiliations:** 1 Department of Pediatric Orthopaedics and Traumatology, Poznan University of Medical Sciences, 61-545 Poznan, Poland; 2 Department of Human Development Psychology and Family Studies, Institute of Psychology, Adam Mickiewicz University, 60-568 Poznan, Poland; 3 Department of Neurosurgery and Neurotraumatology, Poznan University of Medical Sciences, 60-355 Poznan, Poland

**Keywords:** Adolescent idiopathic scoliosis (AIS), Body shape, Brace treatment, Parents, Spinal Appearance Questionnaire

## Abstract

The perspective of trunk deformity is a matter of special concern for adolescent idiopathic scoliosis (AIS) patients. No research group has ever reported interviewing patients and their parents regarding differences in perception of body appearance in the course of Cheneau brace treatment. We aimed to investigate the level of agreement in the field of concerns and perceptions of spinal appearance in relation to brace- and scoliosis-related data between parents and female patients with AIS, treated with a Cheneau brace, by means of the Spinal Appearance Questionnaire-pl (SAQ-pl). In this cross-sectional study forty-one pairs of parents and female patients with AIS were asked to separately complete the Polish versions of the Spinal Appearance Questionnaire-pl patient form (SAQ-pl patient form) and the SAQ-pl parent form. Age of patients was 13.60 years SD 1.60 (range 10–17). Patients scored 2.70 (*SD* 0.60) and parents scored 2.70 *SD* 0.60 in the total score of the SAQ-pl. The study groups do not differ significantly in regards to the SAQ-pl results. The percentage of consistent answers on SAQ-pl items ranges from 34.10 % (item 20) to 78 % (item 8). Height, age and brace-wearing time per day, were significantly related to the differences in the patient-parent General perception of body shape (r_*s*_ = −0.51, r_*s*_ = −0.34, r_*s*_ = 0.36, respectively). Parents and female patients with AIS have similar concerns and perceptions of spinal appearance. The discrepancies in General perception of spinal appearance between parents and AIS females decrease with age of patient. Parental emotional support may contribute to minimizing the risk factors of psychological impairment, especially in late adolescents with AIS.

## Introduction

For most female patients with adolescent idiopathic scoliosis (AIS) the perspective of body deformity is a matter of special concern. Patients observe physical changes in their bodies closely and compare themselves to adolescents without scoliosis, therefore a negative self-evaluation of appearance may lead to different reactions concerning body shape such as body dissatisfaction, negative body image, concern over body size and shape (Dixit et al. 2011). It must be emphasized that adolescence is a period which is especially vulnerable to disturbances in female body image, since in Western societies greater importance of appearance is observed among females (Davison and McCabe 2006; Wardle and Foley 1989).

Moreover, Reichel and Schanz (2003) emphasized that for AIS females brace-wearing represents a major restriction, since it occurs at a time when physical attractiveness is becoming the focus of ever greater attention and is growing in significance. Brace-wearing may also result in greater dependency on the parents, since they have to assist in maintaining the treatment, such as putting on the brace or monitoring wearing times (Braunewell et al. 1987; Freidel 1999). It is important to note that the development of body image is a process influenced by direct and indirect feed-back provided by other people such as peers or family members (McCabe and Ricciardelli 2003; Viviani 2006). Some familial characteristics, for instance negative family attitudes, parental modeling of the importance of physical attractiveness and critical comments about an individual’s physical shape may contribute to negative body image among daughters (Choate 2005). However, parents may provide strong social support for their children by decreasing emphasis on the importance of spinal appearance or body shape in general. A particular role can be ascribed to mothers who can model positive body image and, in this way, teach their daughters to appreciate their bodies (Choate 2005).

Several studies have attempted to use various psychological assessments to define patients’ concerns and assessment of body shape in AIS such as the Scoliosis Research Society-22 (SRS-22; Asher et al. 2003), Scoliosis Research Society-24 (SRS-24; Haher et al. 1999), Short Form-36 (SF-36; McHorney et al. 1994), Quality of Life Profile for Spine Deformities (QLPSD; Climent et al. 2006) and Trunk Appearance Perception Scale (TAPS; Bago et al. 2010; Yang et al. 2013).

Analyses of the differences in patients’ and parents’ perceptions of deformity in AIS by means of the Walter Reed Visual Assessment Scale (WRVAS) or the Spinal Appearance Questionnaire (SAQ), assessment tools comprised of trunk profiles depicting various degrees of trunk deformation, have also been performed (Carreon et al. 2011; Sanders et al. 2007; Sanders et al. 2003; Roy-Beaudry et al. 2011). However, the analyzed groups of patients were mainly under observation or treated operatively. Sanders et al. (2003), having applied the WRVAS, indicated parents perceive deformity of the ribs and shoulders more than the patients, but other aspects of the deformity were identified equally. In another study comparing the scores of patients with those of their parents before surgery (Sanders et al. 2007), parents had significantly worse scores than the patients for the following SAQ domains: Trunk Shift, Kyphosis, Prominence, Shoulders, and Curve.

Based on previous findings, factors associated with the parental and patient evaluation of body shape in adolescent female patients with visible trunk deformities subjected to conservative treatment deserve more attention. In the presented study we aimed to investigate the level of agreement in the field of concerns and perceptions of spinal appearance in relation to brace- and scoliosis-related data between parents and female patients with AIS treated with a Cheneau brace, by means of the SAQ-pl. We hypothesize discrepancies between parents’ and patients’ perceptions of body disfigurement and coexisting associations between brace-wearing duration data and parent-patient disparities in the SAQ scores. As there is no Polish version of the SAQ for parents, one of our objectives was to adapt a method that provides specific data on parents’ perceptions of trunk deformity in AIS patients.

## Method

### Study Design

The study design was cross-sectional. The whole study group was comprised of forty-one female adolescents with idiopathic scoliosis and their parents. We performed an analysis of SAQ scores obtained separately from both parents and patients at the time of the office visit. Mothers accounted for 90.20 % (*n* = 37) of the parent group. All patients were treated conservatively for a minimum of 2 months and were recruited consecutively from one academic centre, the Department and Clinic of Pediatric Orthopaedics and Traumatology at Poznan University of Medical Sciences, by the same doctor, an orthopedic surgeon. The questionnaires were administered during a routine patient visit. All subjects were told about the study upon arrival at the office and they were informed that a refusal to participate in the study would not affect further treatment. The purpose of the study was explained to patients and they were assured that the information they provided would remain anonymous and confidential. The investigator was available throughout the visit in case participants required explanation or clarification.

### Study Participants

The selection criteria were the following: a minimum duration of Cheneau brace application of at least 12 h a day, a Cobb angle of 20–40°, 10–17 years of age at the time of selection to the study. The data concerning brace-wearing compliance was based on interviews with patients and parents. The criterion of recommendation for initiating brace treatment, concerning skeletal maturity (Risser sign from 0 to 2), was based on a study by Richards et al. (2005). We excluded patients suffering from other diseases leading to trunk deformity or other serious medical conditions. 63.4 % of patients and parents (n = 26) lived in an urban area, whereas 36.6 % of patients and parents (n = 15) were from a rural area.

In the patient group, 56.10 % had thoracic scoliosis, 34.10 % had thoraco-lumbar scoliosis, and the remaining 9.10 % of patients had lumbar scoliosis. Th8 was the apical vertebra in eleven patients; Th9 in three patients; Th10 in four patients; Th11 in six patients; and Th12 in five patients. L1 was the apical vertebra in eight patients; L2 in two, and L3 in two patients.

After grouping the subjects based on the second quartile (median) distribution of the results by “time in brace”, we indicated 21 female patients (51.20 %) wore the brace for 2–12 months, whereas 20 females (48.80 %) wore the brace for >12 months. Furthermore, daily brace-wearing data was split into two categories: 32 females (78 %) wore the brace from 12 h to 17 h a day and 9 females (22 %) wore the brace from 18 to 22 h a day. For detailed characteristics of the female scoliosis patients (see Table 1).

**Table 1:** Clinical and socio-demographic patient characteristics

Parameters	Mean	Range		SD
		Min.	Max.	
Weight (kg)	47.80	26.00	62.00	8.10
Height (cm)	161.60	142.00	175.00	6.90
Body mass index	18.20	12.90	22.90	2.30
Age (years)	13.60	10.00	17.00	1.60
Cobb angle	27.60	20.00	40.00	5.70
Angle of trunk rotation^a^	6.40	1.00	13.00	3.50
Apical translation (cm)^b^	1.90	0.40	4.00	1.00
Brace (hours/day)	16.10	12.00	22.00	2.40
Brace (in months)	17.00	2.00	58.00	14.90

^a^Angle of trunk rotation as measured with Perdriolli’s inclinometer; ^b ^The degree of the apical translation of center sacral vertical line (CSVL) according to the Harms Study Group

### Ethical Issues

All study participants received detailed information on the aim of the study and were assured of anonymity after which they gave their informed consent. The study was approved by the Bioethics Committee.

### Adaptation Procedure

All patients completed the Polish version of SAQ (SAQ-pl) for patients (Misterska et al. 2011). As there is no SAQ-pl for parents, we adapted this questionnaire to Polish cultural settings. This process was compliant with the guidelines of the International Quality of Life Assessment (IQOLA) Project (Beaton et al. 2000). Thirty-seven parents filled out the final version of the SAQ-pl parent form twice following a 2 day interval.

### Assessment of Spinal Deformity

The SAQ was developed from the Walter Reed Visual Assessment Scale, which contains images of trunk profiles depicting various degrees of trunk deformity caused by scoliosis only. The SAQ instrument is divided into 2 sections: the first relies on drawings adapted from the WRVAS, and the second contains textual questions rating dissatisfaction with other aspects of spinal deformity appearance.

The SAQ has been supported as reliable, valid, and responsive. The individual scale items have been recorded as having good to excellent test–retest reliability (Spearman’s rho, 0.57–0.99) for patients and parents. Within each scale, there was high internal consistency (Cronbach’s alpha > 0.7). In addition, the SAQ provides more detail than the SRS appearance domain and clearer explanation of concerns about spinal deformity and improvements. However, it must be emphasized, the previous testing of WRVAS figures which were incorporated into the SAQ, indicated the profile of the individual WRVAS scores failed to differentiate between specific curve patterns (thoracic, double major and thoracolumbar/lumbar). In addition, some figures, such as flank prominence, trunk imbalance and shoulder asymmetry, did not correlate with the radiological deformity they were designed to measure (Pineda et al. 2006). Sanders et al. (2007) indicated the SAQ domain scores correlate with curve magnitude and appear to measure patients’ perceptions of different aspects of their curves. The SAQ also demonstrates excellent responsiveness to surgical curve correction. The differing scales appear to correlate with the different components of scoliosis as should be expected, indicating that the scale identifies clinically evident problems and that the patients and their parents notice these issues. However, the least responsive SAQ domain to surgery was the Chest. In addition, Mulcahey et al. (2011) pointed out both typically-developing youth and those with idiopathic scoliosis experience problems in understanding every written and pictorial SAQ item, due to complex medical words, vague questions and difficult illustrations. In particular, subjects had difficulty understanding the meaning of specific words such as “prominence” and “flank”. The pictorial illustrations for items 2 and 3, 4 and 5 were problematic for investigated adolescents.

The SAQ for patients consists of 20 items, whereas the SAQ- parent form consists of 21 items. The additional question regards the postoperative scar where the parent is asked how, versus the patient, he/she views the appearance of the surgical scar. These items form the following nine subscales: General, Curve, Prominence, Trunk shift, Waist, Shoulders, Kyphosis, Chest and Surgical scar. Our study applies to patients treated conservatively only, therefore we omitted the Surgical scar domain in the analyses. Questions no. 8, 18 and 20 are open-ended questions that focus on which aspect of deformity is the most bothersome to patients. Question no. 8 reads *Which form of deformity bothers you the most out of these 5 categories of images?*, question no. 18 *Of questions* 9–17, *which are the most important to you?*, whereas question no. 20 reads *What would you most like to change about your body shape?* The remaining items are scored from *1* (best) to *5* points (worst). Each domain as well as total score are usually expressed as the average of all item responses and, therefore, the range is from 1 to 5 points. As scores increase so the assessment of patients’ spinal appearance worsens (Bago et al. 2007; Pineda et al. 2006; Sanders et al. 2003; Sanders et al. 2007).

### Statistical Methods

In respect to statistical quantitative features, we determined mean, 95 % confidence intervals, range and standard deviations. Concerning qualitative features, we assigned respective percentages to the number of units that belong to described categories of a given feature. In regards to SAQ-pl for parents, we analyzed the percentage of subjects scoring the minimum (floor effect) and maximum (ceiling effect). To assess internal consistency, we used Cronbach’s alpha. Values of Cronbach’s alpha coefficients above 0.80 were considered as excellent (Salter et al. 2005). The assessment of the test–retest reliability was performed using the Intraclass Correlation Coefficients (ICCs). ICC values above 0.80 were considered as evidence of excellent reliability (Nunnally and Bernstein 1994). We used Wilcoxon signed ranks tests to compare patients’ and parents’ perceptions of body appearance. Spearman’s rank order correlation coefficients were applied to evaluate correlations between quantitative variables. Cohen’s kappa coefficient was used to measure the agreement between the items on the SAQ-pl parent and patient forms. We considered Cohen’s kappa coefficients above 0.40 as good agreement (Landis and Koch 1977).

The logistic regression analysis was applied to define the degree to which trunk deformity affects perception of spinal appearance by parents and female patients with AIS. Based on the lower and upper quartile distribution of the SAQ-pl total score, expressed as the sum of the closed-ended questions, the results were split into two categories: “good result” (from 16 to 32 points) and “poor result” (above 32 points). We evaluated the influence of the socio-demographic, brace-related and radiological data, on the probability of achieving a “good result” in the SAQ questionnaire. We set the border level of statistical significance at *p* = 0.05; test results whose *p* value exceeded this level were treated as insignificant. We performed statistical calculations by means of Statistica software.

## Results

### Descriptive Statistics

In the patient subgroup, the mean value of the SAQ total score was 2.70 *SD* 0.60. Patients exhibit the most self-criticism in the following order: General, Waist, Shoulders, Chest, Curve, Trunk shift and Kyphosis. Meanwhile, Prominence was the element that was assessed the least critically by patients. In the parent sample the mean value of the SAQ total score was 2.70 *SD* 0.60. Parents exhibit the most criticism in the following order: General, Waist, Chest, Shoulders, Curve. Trunk shift, Kyphosis and Prominence were the least criticized elements of body shape in patients by the parents (for details see Table 2).

**Table 2:** Distribution of results of SAQ-pl parents form and SAQ-pl patients form

SAQ-pl subscales	Mean	95 % CI		Min.	Max.	SD	Mean	95 % CI		Min.	Max.	SD	*p* value
		From	To					From	To				
	Patients						Parents						
General	3.5	3.3	3.8	1.3	5.0	0.8	3.5	3.3	3.8	1.0	4.7	0.7	0.846
Curve	2.4	2.2	2.6	1.0	4.0	0.7	2.3	2.1	2.5	1.0	4.0	0.6	0.142
Prominence	1.7	1.6	1.9	1.0	2.5	0.4	1.9	1.7	2.0	1.0	3.0	0.5	0.059
Trunk shift	1.9	1.7	2.0	1.0	3.0	0.5	1.9	1.7	2.1	1.0	3.5	0.5	0.510
Waist	3.4	3.0	3.8	1.0	5.0	1.3	3.3	2.8	3.7	1.0	5.0	1.4	0.326
Shoulders	2.8	2.5	3.2	1.0	5.5	1.0	2.8	2.4	3.1	1.0	4.0	1.0	0.673
Kyphosis	1.9	1.6	2.1	1.0	3.0	0.7	1.9	1.6	2.1	1.0	3.0	0.7	1.000
Chest	2.6	2.2	3.1	1.0	5.0	1.5	2.9	2.2	3.3	1.0	5.0	1.7	0.581
Total score	2.7	2.5	2.9	1.3	3.8	0.6	2.7	2.5	2.9	1.0	3.9	0.6	0.343

From the interpretation of the answers given to question 8, it seems that the head-rib-pelvic alignment is the most disturbing issue of AIS, both for the patient and parent subgroups (29.30 and 39 %, respectively). Considering the answers given to question 18, it seems that as many as 65.90 % of the parent group and 58.50 % of patients would rather have a straighter shape. The distribution of answers to question 20 revealed that as many as 58.50 % of parents and 48.80 % of patients would change body shape in general. Moreover, only 4.90 % of parents and none of the patients would not change anything in body shape.

### Psychometric Properties of the SAQ-pl parent form

The Cronbach’s alpha value of the general result of SAQ-parent form was excellent and equaled 0.81 (95 % CI 0.708–0.880) in the test and 0.83 (95 % CI 0.727–0.896) in the retest. Similarly, the test–retest reliability was excellent and equaled 0.99 (95 % CI 0.988–0.997). There were no floor or ceiling effects, regarding the total score of SAQ. However, a moderate floor effect was observed in 5 domains: Trunk shift (9.80 %), Waist (9.80 %), Shoulders (14.60 %), Kyphosis (29.30 %) and Chest (31.70 %). Meanwhile, a moderate ceiling effect was observed in the following two domains of the SAQ-pl parent form: Waist (19.50 %) and Chest (29.30 %).

### Comparative Analyses

The analysis revealed patient and parent subgroups did not differ significantly in regards to the total score (*p* > 0.05) and the individual domains of the SAQ-pl (*p* > 0.05).

We estimated the percentage of consistent answers on SAQ-pl items both for the female patients with AIS and their parents, to assess the agreement between parents’ and patients’ perceptions of spinal appearance. The consistency ranges from 34.10 % (item 20) to 78 % (item 9). Cohen’s kappa coefficients were calculated except for the open-ended questions and range from 0.115 (item 6) to 0.538 (item 1), indicating poor to good agreement between parents’ and patients’ assessment of body shape. The highest agreement, above the value of 0.40, regards items 1, 2, 7, 13 and 15, from the Curve, Prominence, Waist, Kyphosis and Chest domains, respectively.

Having analyzed the associations between SAQ-pl parent and patient results, we indicated a significant positive correlation between patient/parental assessment of General, Curve, Prominence, Trunk shift, Waist, Shoulders, Kyphosis, Chest and in the total score (r_*s*_ = 0.52, r_*s*_ = 0.69, r_*s*_ = 0.40, r_*s*_ = 0.58, r_*s*_ = 0.50, r_*s*_ = 0.55, r_*s*_ = 0.55, r_*s*_ = 0.62). For details see Table 3.

**Table 3:** Correlation between results of SAQ-pl parent and SAQ-pl patient forms

SAQ-pl patient form									
Domains	General	Curve	Prominence	Trunk shift	Waist	Shoulders	Kyphosis	Chest	Total score
*SAQ-pl parent form*									
General	r_*s*_ = 0.52*	r_*s*_ = 0.26	r_*s*_ = 0.16	r_*s*_ = 0.05	r_*s*_ = 0.40*	r_*s*_ = 0.30	r_*s*_ = 0.04	r_*s*_ = 0.37*	r_*s*_ = 0.43*
Curve	r_*s*_ = 0.39*	r_*s*_ = 0.69*	r_*s*_ = 0.03	r_*s*_ = 0.32*	r_*s*_ = 0.32*	r_*s*_ = 0.45*	r_*s*_ = 0.15	r_*s*_ = 0.13	r_*s*_ = 0.48*
Prominence	r_*s*_ = 0.29	r_*s*_ = 0.09	r_*s*_ = 0.40*	r_*s*_ = 0.51*	r_*s*_ = 0.24	r_*s*_ = 0.35*	r_*s*_ = 0.27	r_*s*_ = 0.18	r_*s*_ = 0.41*
Trunk shift	r_*s*_ = 0.36*	r_*s*_ = 0.33*	r_*s*_ = 0.01	r_*s*_ = 0.25	r_*s*_ = 0.29	r_*s*_ = 0.35*	r_*s*_ = 0.26	r_*s*_ = 0.36*	r_*s*_ = 0.43*
Waist	r_*s*_ = 0.56*	r_*s*_ = 0.06	r_*s*_ = 0.06	r_*s*_ = 0.18	r_*s*_ = 0.58*	r_*s*_ = 0.19	r_*s*_ = 0.02	r_*s*_ = 0.19	r_*s*_ = 0.42*
Shoulders	r_*s*_ = 0.51*	r_*s*_ = 0.13	r_*s*_ = 0.02	r_*s*_ = 0.09	r_*s*_ = 0.48*	r_*s*_ = 0.50*	r_*s*_ = 0.17	r_*s*_ = 0.48*	r_*s*_ = 0.58*
Kyphosis	r_*s*_ = 0.35*	r_*s*_ = 0.39*	r_*s*_ = 0.07	r_*s*_ = 0.16	r_*s*_ = 0.13	r_*s*_ = 0.13	r_*s*_ = 0.55*	r_*s*_ = 0.23	r_*s*_ = 0.31
Chest	r_*s*_ = 0.29	r_*s*_ = 0.15	r_*s*_ = 0.15	r_*s*_ = −0.06	r_*s*_ = 0.25	r_*s*_ = 0.34*	r_*s*_ = 0.13	r_*s*_ = 0.55*	r_*s*_ = 0.41*
Total score	r_*s*_ = 0.62*	r_*s*_ = 0.25	r_*s*_ = 0.15	r_*s*_ = 0.13	r_*s*_ = 0.56*	r_*s*_ = 0.43*	r_*s*_ = 0.17	r_*s*_ = 0.49*	r_*s*_ = 0.62*

* *p* < 0.05; SAQ-pl-Polish version of the SAQ

### Associations Between SAQ-pl Results and Patient Characteristics

Having analyzed the correlation between patient characteristics and SAQ-pl parent results, we indicated a correlation between: General parental assessment of body shape and duration of brace wearing a day (r_*s*_ = 0.33), assessment of Curve and Cobb angle (r_*s*_ = 0.40) and duration of brace wearing in months (r_*s*_ = −0.33), assessment of Prominence and apical translation (r_*s*_ = 0.44), perception of Trunk shift and Cobb angle (r_*s*_ = 0.31) and perception of Chest and duration of brace wearing a day (r_*s*_ = 0.37) (Table 4).

**Table 4:** Correlation between SAQ-pl parent results and patients characteristic

SAQ-pl parent form	General	Curve	Prominence	Trunk shift	Waist	Shoulders	Kyphosis	Chest	Total score
Weight	r_*s*_ = −0.03	r_*s*_ = −0.07	r_*s*_ = 0.18	r_*s*_ = −0.16	r_*s*_ = 0.04	r_*s*_ = 0.03	r_*s*_ = −0.10	r_*s*_ = −0.17	r_*s*_ = −0.01
Height	r_*s*_ = −0.25	r_*s*_ = −0.10	r_*s*_ = 0.04	r_*s*_ = −0.06	r_*s*_ = 0.04	r_*s*_ = 0.08	r_*s*_ = 0.01	r_*s*_ = −0.20	r_*s*_ = −0.05
Body mass index	r_*s*_ = 0.09	r_*s*_ = 0.02	r_*s*_ = 0.23	r_*s*_ = −0.18	r_*s*_ = −0.03	r_*s*_ = −0.04	r_*s*_ = −0.13	r_*s*_ = −0.12	r_*s*_ = −0.03
Age	r_*s*_ = −0.19	r_*s*_ = 0.10	r_*s*_ = −0.06	r_*s*_ = −0.06	r_*s*_ = 0.01	r_*s*_ = −0.01	r_*s*_ = 0.05	r_*s*_ = −0.32*	r_*s*_ = −0.14
Cobb angle	r_*s*_ = −0.05	r_*s*_ = 0.40*	r_*s*_ = 0.12	r_*s*_ = 0.31*	r_*s*_ = 0.02	r_*s*_ = 0.18	r_*s*_ = 0.22	r_*s*_ = −0.02	r_*s*_ = 0.09
Angle of trunk rotation	r_*s*_ = −0.26	r_*s*_ = −0.03	r_*s*_ = 0.02	r_*s*_ = 0.13	r_*s*_ = −0.17	r_*s*_ = 0.19	r_*s*_ = −0.05	r_*s*_ = 0.14	r_*s*_ = −0.04
Apical translation	r_*s*_ = 0.08	r_*s*_ = 0.15	r_*s*_ = 0.44*	r_*s*_ = 0.14	r_*s*_ = 0.21	r_*s*_ = 0.12	r_*s*_ = 0.18	r_*s*_ = 0.01	r_*s*_ = 0.19
Brace (hours/day)	r_*s*_ = 0.33*	r_*s*_ = 0.12	r_*s*_ = −0.10	r_*s*_ = 0.19	r_*s*_ = 0.02	r_*s*_ = −0.01	r_*s*_ = 0.04	r_*s*_ = 0.37*	r_*s*_ = 0.22
Brace (in months)	r_*s*_ = −0.01	r_*s*_ = −0.33*	r_*s*_ = −0.01	r_*s*_ = −0.16	r_*s*_ = 0.18	r_*s*_ = −0.19	r_*s*_ = −0.01	r_*s*_ = −0.04	r_*s*_ = −0.02

* *p* < 0.05; SAQ-pl-Polish version of the SAQ

Analysis of the relations between SAQ-pl patient results and patient characteristics revealed that the only significant correlations regard the perception of Prominence and apical translation (r_*s*_ = 0.31), assessment of Trunk shift and Cobb angle and Trunk shift and apical translation (r_*s*_ = 0.31 and r_*s*_ = 0.36, respectively) (Table 5).

**Table 5:** Correlation between SAQ-pl parent-patient differences and patients characteristics

SAQ-pl parent-patient differences	General	Curve	Prominence	Trunk shift	Waist	Shoulders	Kyphosis	Chest	Total score
Weight	r_*s*_ = −0.26	r_*s*_ = 0.01	r_*s*_ = 0.11	r_*s*_ = −0.13	r_*s*_ = 0.04	r_*s*_ = 0.06	r_*s*_ = −0.08	r_*s*_ = −0.06	r_*s*_ = 0.02
Height	r_*s*_ = −0.51*	r_*s*_ = 0.15	r_*s*_ = 0.20	r_*s*_ = 0.02	r_*s*_ = 0.11	r_*s*_ = 0.08	r_*s*_ = −0.08	r_*s*_ = −0.07	r_*s*_ = 0.06
Body mass index	r_*s*_ = −0.04	r_*s*_ = −0.09	r_*s*_ = 0.05	r_*s*_ = −0.21	r_*s*_ = −0.12	r_*s*_ = −0.04	r_*s*_ = −0.02	r_*s*_ = −0.09	r_*s*_ = −0.09
Age	r_*s*_ = −0.34*	r_*s*_ = 0.22	r_*s*_ = 0.13	r_*s*_ = −0.04	r_*s*_ = 0.07	r_*s*_ = 0.10	r_*s*_ = 0.26	r_*s*_ = −0.10	r_*s*_ = 0.09
Cobb angle	r_*s*_ = −0.02	r_*s*_ = 0.07	r_*s*_ = −0.02	r_*s*_ = 0.04	r_*s*_ = 0.11	r_*s*_ = 0.09	r_*s*_ = 0.07	r_*s*_ = 0.13	r_*s*_ = 0.11
Angle of trunk rotation	r_*s*_ = −0.26	r_*s*_ = −0.37*	r_*s*_ = −0.06	r_*s*_ = −0.04	r_*s*_ = −0.15	r_*s*_ = 0.09	r_*s*_ = −0.17	r_*s*_ = 0.07	r_*s*_ = −0.19
Apical translation	r_*s*_ = −0.06	r_*s*_ = 0.24	r_*s*_ = 0.17	r_*s*_ = −0.12	r_*s*_ = 0.10	r_*s*_ = 0.11	r_*s*_ = 0.09	r_*s*_ = 0.09	r_*s*_ = 0.18
Brace (hours/day)	r_*s*_ = 0.36*	r_*s*_ = −0.01	r_*s*_ = −0.18	r_*s*_ = 0.21	r_*s*_ = 0.19	r_*s*_ = −0.12	r_*s*_ = −0.03	r_*s*_ = 0.24	r_*s*_ = 0.18
Brace (in months)	r_*s*_ = 0.03	r_*s*_ = −0.07	r_*s*_ = −0.04	r_*s*_ = −0.22	r_*s*_ = 0.13	r_*s*_ = 0.02	r_*s*_ = −0.16	r_*s*_ = 0.10	r_*s*_ = 0.05

* *p* < 0.05

We have analyzed the relation between differences in patient-parent SAQ-pl results and patient characteristics. To analyze if a younger age led to disparities between parents’ and patients’ perceptions of spinal appearance, Spearman’s correlations of patients’ age with differences in patient–parent SAQ scores were performed. Correlations of Cheneau brace application (daily and monthly) and clinical and radiographic scoliosis parameters with differences in patient–parent SAQ scores were applied to verify if patient–parent perspectives correlate better as bracing time or severity of spinal deformity increases. This analysis revealed significant but moderate and weak correlations between the patient-parent differences in the General perception of body shape and height (r_*s*_ = −0.51, *p* < 0.05), age (r_*s*_ = −0.34, *p* < 0.05), and the duration of brace-wearing per day (r_*s*_ = 0.36, *p* < 0.05). Moreover, the angle of trunk rotation was found to be related to the patient-parent differences in perception of the Curve domain (r_*s*_ = −0.37, *p* < 0.05) (see Table 6).

**Table 6:** Correlation between SAQ-pl parent-patient differences and patient characteristics

SAQ-pl parent-patient differences	General	Curve	Prominence	Trunk shift	Waist	Shoulders	Kyphosis	Chest	Total score
Weight	r_*s*_ = −0.26	r_*s*_ = 0.01	r_*s*_ = 0.11	r_*s*_ = −0.13	r_*s*_ = 0.04	r_*s*_ = 0.06	r_*s*_ = −0.08	r_*s*_ = −0.06	r_*s*_ = 0.02
Height	r_*s*_ = −0.51*	r_*s*_ = 0.15	r_*s*_ = 0.20	r_*s*_ = 0.02	r_*s*_ = 0.11	r_*s*_ = 0.08	r_*s*_ = −0.08	r_*s*_ = −0.07	r_*s*_ = 0.06
Body mass index	r_*s*_ = −0.04	r_*s*_ = −0.09	r_*s*_ = 0.05	r_*s*_ = −0.21	r_*s*_ = −0.12	r_*s*_ = −0.04	r_*s*_ = −0.02	r_*s*_ = −0.09	r_*s*_ = −0.09
Age	r_*s*_ = −0.34*	r_*s*_ = 0.22	r_*s*_ = 0.13	r_*s*_ = −0.04	r_*s*_ = 0.07	r_*s*_ = 0.10	r_*s*_ = 0.26	r_*s*_ = −0.10	r_*s*_ = 0.09
Cobb angle	r_*s*_ = −0.02	r_*s*_ = 0.07	r_*s*_ = −0.02	r_*s*_ = 0.04	r_*s*_ = 0.11	r_*s*_ = 0.09	r_*s*_ = 0.07	r_*s*_ = 0.13	r_*s*_ = 0.11
Angle of trunk rotation	r_*s*_ = −0.26	r_*s*_ = −0.37*	r_*s*_ = −0.06	r_*s*_ = −0.04	r_*s*_ = −0.15	r_*s*_ = 0.09	r_*s*_ = −0.17	r_*s*_ = 0.07	r_*s*_ = −0.19
Apical translation	r_*s*_ = −0.06	r_*s*_ = 0.24	r_*s*_ = 0.17	r_*s*_ = −0.12	r_*s*_ = 0.10	r_*s*_ = 0.11	r_*s*_ = 0.09	r_*s*_ = 0.09	r_*s*_ = 0.18
Brace (hours/day)	r_*s*_ = 0.36*	r_*s*_ = −0.01	r_*s*_ = −0.18	r_*s*_ = 0.21	r_*s*_ = 0.19	r_*s*_ = −0.12	r_*s*_ = −0.03	r_*s*_ = 0.24	r_*s*_ = 0.18
Brace (in months)	r_*s*_ = 0.03	r_*s*_ = −0.07	r_*s*_ = −0.04	r_*s*_ = −0.22	r_*s*_ = 0.13	r_*s*_ = 0.02	r_*s*_ = −0.16	r_*s*_ = 0.10	r_*s*_ = 0.05

* *p* < 0.05; SAQ-pl-Polish version of the SAQ

After grouping the subjects by “time in brace”, concerning daily duration of brace-wearing, we revealed that patients from both study groups do not differ in regards to SAQ scores, however, parents of female AIS patients differ significantly in regards to perception of Chest (*p* = 0.044, parents of female AIS patients wearing the brace from 12 to 17 h per day perceived patients’ spinal appearance less critically. After analysis concerning duration of orthosis-wearing in months we indicated parents of patients from both study groups differ in regards to the Curve domain (*p* = 0.032, parents of AIS patients wearing the brace from 2 to 12 months perceive patients’ body shape more critically), whereas patients do not differ significantly in regards to SAQ scores.

### Regression Analysis

The logistic regression model obtained as a result of the calculations revealed that age, BMI, Cobb angle, angle of trunk rotation, apical translation and duration of brace wearing, do not have a statistically significant influence on the probability of achieving a good result in the SAQ-pl in either study group.

## Discussion

The aim of the current study was to investigate patients’ and parents’ perceptions of appearance in scoliosis treated with a brace in the Polish population. Therefore, whilst the presented findings may not be generalizable across other countries they provide further insight into spinal appearance issues in AIS females from a developing country such as Poland where the social situation of the disabled is substantially different compared to developed society (Gąciarz et al. 2008). When considering the possibly crucial differences in the study results and condition of patients between this Polish sample and samples from developed countries, we should take into account the economic, social and political changes in Polish society which were introduced after the fall of the Communist regime. In developing countries, such as Poland, there is often a noticeable economic imbalance in different regions. In comparison to rural areas, life-style in urban regions may seem relatively open, with a higher income per capita and better quality of health care system standards. In addition, the aspects central to government policy towards the disabled are usually only implemented in some urban centers. It is not possible therefore to speak of a comprehensive public policy for the disabled in Poland. In many families in Poland, a disabled child is regarded as a cause for shame and should be hidden from the community. This does not aid improvement in the social environment or help raise public awareness of the issue. Social exclusion affects the disabled from rural regions to a greater degree than the disabled from urban areas (Gąciarz et al. 2008).

Many authors emphasize that brace-wearing for AIS females represents a major restriction, since it takes place at time when physical attractiveness is becoming increasingly significant (Reichel and Schanz 2003). Brace-wearing may also result in greater dependency on the parents, since they have to assist in maintaining the treatment, such as putting on the brace or monitoring wearing times (Braunewell et al. 1987; Freidel 1999).

In the current study design we focused on the agreement and discrepancies in parents’ and patients’ perceptions of appearance. We demonstrated that the General perception of body shape and assessment of waist asymmetry were the elements most critically assessed by both patients and parents. Our study was applied to patients from a homogenous group of AIS females, treated conservatively with Thoraco-Lumbar Sacral Orthosis (TLSO), only. We are not aware of any previous study that has compared the SAQ scores with duration of brace-wearing data.

As mentioned, all patients completed the Polish version of SAQ-pl for patients, which was published in 2011 (Misterska et al. 2011). Along with this research project, which started in 2009, we were gathering material for the current research, accounting for the possible discrepancies in parents’ and patients’ perceptions of spinal appearance. We did not use the later version of the SAQ, published by Carreon et al. in 2011 (Carreon et al. 2011) for this reason. In a previous study concerning the French-Canadian version of the SAQ for patients (SAQ-fv), the authors did not conduct an analysis of the Cronbach’s alpha and ICCs for the SAQ-fv. Moreover, differences between the SAQ-fv for parents and patients were not explored (Roy-Beaudry et al. 2011). Meanwhile, we confirmed excellent internal consistency and test–retest reliability of the SAQ-pl for parents.

We took different indicators of consistency between parents and patients into consideration, such as the percentage of consistent answers, Cohen’s coefficient and Spearman’s correlation coefficients and revealed similar concerns and perceptions of body shape in both study groups. However, the highest consistency applies to the perception of Curve, Prominence, Waist, Kyphosis and Chest. Interestingly, Sanders et al. (2007) indicated that parents had significantly worse scores than patients treated operatively for the following domains of the SAQ: Trunk shift, Kyphosis, Prominence, Shoulders, and Curve. In another study (Sanders et al. 2003), it was reported that parents perceive a higher degree of deformity, measured by WRVAS, of the ribs and shoulders than the patients, but other aspects of the deformity are identified equally. In contrast, our study indicated both parents and AIS patients perceive all investigated forms of spinal deformity, measured by SAQ, equally.

In particular, we demonstrated the relation between age and patient-parent differences in the General subscale of the SAQ (r_*s*_ = −0.34), signifying that the difference in the General perception of spinal appearance subscale between parents and patients declines as age of patient increases. This pattern is consistent with developmental psychologists’ claims that late adolescents, although being critical towards adults and absorbed with peer relations, increasingly identify with their parents’ values. Therefore, parents of late adolescents may be a significant and supportive source of evaluation of spinal appearance, in comparison with parents of early adolescents, whose perception of body shape may be influenced to a greater extent by peers (Boyd and Bee 2004).

Interestingly, the analysis of associations between parents’ perception of spinal appearance and brace-related data revealed the negative influence of duration of brace-wearing per day, but also the positive role of long-term orthosis-wearing measured in months as well as, most probably, the outcome of brace-wearing, on parental adaptation to AIS conservative treatment.

There are some limitations in this cross-sectional study that should be pointed out. It is necessary to underline that only female patients were investigated in the study, which limits the generalizability of the presented findings. Furthermore, we did not aim to evaluate the effectiveness of the conservative treatment on AIS patient and parental satisfaction and perception of spinal appearance. Therefore, future research would benefit from longitudinal assessment of body disfigurement by means of SAQ from the perspective of male and female scoliosis patients and both parents, in the course of operative or conservative treatment of AIS. Moreover, we suggest a separate analysis of the perceptions of mothers versus fathers concerning the spinal appearance of patients with AIS in order to achieve better homogeneity of the study group. In addition, analysis of other parent-related data, such as age, occupation, or educational level, would help us better understand the investigated patient-parent disparities.

Considering the practical implications, the possibilities for psychological intervention for AIS patients may concern parental support, since their emotional support and attempts to decrease the emphasis on the importance of body shape can contribute to minimizing the risk factors involved in body image disturbances and other psychological impairment in AIS patients (Freidel 1999).

In conclusion, the key-findings are that parents and female patients with AIS have similar concerns and perceptions of spinal appearance. Differences in the General perception of spinal appearance subscale between parents and patients decrease as age of patient increases. Cobb angle and duration of brace-wearing per day and in months affect parents’ perceptions of AIS patients’ spinal appearance, whereas SAQ scores are influenced by Cobb angle. Parental emotional support, as a significant and supportive source of evaluation of spinal appearance, may contribute to minimizing the risk factors of psychological impairment, especially in late adolescents with AIS.
